# The Predicting Role of Serum TSGF and sIL-2R for the Lymph Node Metastasis of Papillary Thyroid Carcinoma

**DOI:** 10.1155/2022/3730679

**Published:** 2022-09-02

**Authors:** Xiaoqin Xu, Weigang Wang, Ting Sun, Baoguo Tian, Lili Du, Jiexian Jing

**Affiliations:** ^1^Department of Etiology, Shanxi Province Cancer Hospital, Taiyuan, Shanxi, China; ^2^Shanxi Hospital Affiliated to Cancer Hospital, Chinese Academy of Medical Sciences, Taiyuan, Shanxi, China; ^3^Cancer Hospital Affiliated to Shanxi Medical University, Taiyuan, Shanxi, China

## Abstract

**Objective:**

To explore the clinical utility of tumor-specific growth factor (TSGF) and the soluble interleukin-2 (IL-2) receptor (sIL-2R) as immune-related factors for predicting lymph node metastases (LNM) of papillary thyroid carcinoma (PTC).

**Methods:**

A total of 206 patients with PTC subjected to curative surgery were enrolled. All patients had complete medical records. Serum levels of TSGF were detected using Automatic Biochemistry Analyzer and the serum sIL-2R concentration was detected by enzyme-linked immunosorbent assay (ELISA). Furthermore, we analyzed the relationship between the two indicators and the clinicopathological characteristics and assessed their effect on lymphatic metastasis in patients with PTC by logistic regression analysis.

**Results:**

Receiver operating characteristic (ROC) analysis revealed that the determined cut-off value of serum TSGF and sIL-2R was 63.35 U/mL and 507 U/mL, respectively. Serum TSGF was associated with focality (*χ*^2^ = 4.97, *P* = 0.026) and lymphatic metastasis (*χ*^2^ = 4.154, *P* = 0.042), while serum sIL-2R was remarkably related to gender (*χ*^2^ = 4.464, *P* = 0.035). Univariate logistic regression analysis indicated that age, tumor size, serum TSGF level, capsule invasion, and nodular goiter were the lymphatic metastasis-related factor of PTC. Multivariate regression analysis revealed that age > 45 years was a protective factor (OR: 0.4, 95% CI: 0.206-0.777, *P* = 0.007). Conversely, larger tumor size (OR: 4.594, 95% CI: 2.127-9.921, *P* = 0.000), higher serum TSGF levels (OR: 1.888, 95% CI: 1.009-3.533, *P* = 0.047), and capsule invasion (OR: 1.939, 95% CI: 1.009-3.726, *P* = 0.047) were associated with an increased risk of LNM.

**Conclusion:**

Serum TSGF levels were identified as an independent factor for LNM in patients with PTC.

## 1. Introduction

Thyroid cancer is the most common malignancies affecting the endocrine system, with an increased incidence due to early detection, a higher prevalence of modifiable individual risk factors, and high exposure to environmental risk factors [1]. Papillary thyroid carcinoma (PTC) is the predominant histological subtype and is characterized by expanded occurrence and lymph node metastasis (LNM). Although it rarely leads to disease-specific mortality, the identification of the individuals that are at increased risk for the development of thyroid cancer will improve the clinical management of PTC.

Tumor-specific growth factor (TSGF) and the soluble interleukin-2 (IL-2) receptor (sIL-2R) are immune-related factors. To date, increasing evidence has shown diverse roles for TSGF in the diagnosis and evaluation of therapeutic effect assessment in different cancers including breast cancer [2, 3], gastric cancer [4], pancreatic cancer [5, 6], colon cancer [7], bladder cancer [8], prostate cancer [9], hepatocellular carcinoma [10], carcinoma of the endometrium [11], oral squamous cell carcinoma [12], osteosarcoma [13], renal cancer [14], and nasopharyngeal carcinoma [15]. Of these, TSGF combined with traditional biomarkers displayed important clinic applicability. Color Doppler ultrasound combined with serum levels of cancer antigen 15-3 (CA15-3), carcinoembryonic antigen (CEA), and TSGF improved the diagnostic accuracy of breast cancer [2]. The imaging accuracy of TSGF and tumor marker combined with 18F-FDG-PET is approximately 97.3% and achieves 100% specificity for the diagnosis of prostate cancer [9]. The combined assay of serum CEA, cancer antigen 72-4 (CA72-4), cancer antigen 19-9 (CA19-9), and TSGF has shown promising discriminatory power in gastric cancer (AUC = 0.913, sensitivity: 88.9%) [4], while serum levels of TSGF, cancer antigen 242 (CA242), and CA19-9 for the identification of pancreatic cancer [5] and serum levels of AC007271.3, squamous cell carcinoma antigen (SCCA), and TSGF for oral squamous cell carcinoma resulted in an AUC of 0.917 with a sensitivity of 80.0% and specificity of 93.1% [12]. TSGF can predict the outcome of pancreatic cancer after cryoablation treatment [6], can be used to monitor the treatment effect in colon cancer [7], and can help determine the outcome of robot-assisted radical cystectomy in bladder cancer [8]. Primary hepatocellular carcinoma patients with high TSGF expression achieved a low 3-year survival rate and predicted the curative effects of transcatheter arterial chemoembolization (TACE) [10]. Serum TSGF levels were associated with a 5-year survival rate in patients with osteosarcoma [13]. Altogether these findings indicate that serum TSGF exerts an important role in the identification and the prognosis and therapeutic outcome of cancer. However, its roles in PTC have not been elucidated.

sIL-2R was first reported in activated peripheral blood T cell supernatants in 1985 [16]. sIL-2R was considered as a marker of T-lymphocyte activation, increasing in many malignancies, including thyroid cancer. According to a previous study, the levels of sIL-2R were positively associated with free thyroxine (FT4)/free triiodothyronine (FT3) [17], which was modifiable in a thyroid function-dependent manner following postoperative L-thyroxine (L-T4) therapy after surgery and disease progression [18, 19]. Furthermore, the role of sIL-2R in carcinogenesis and development has been extensively studied, including ovarian cancer [20], diffuse large B cell lymphoma [21], familial breast cancer [22], and lung cancer [23]. Besides, the serum sIL-2R/ferritin ratio is a promising candidate to detect lymphoma-associated hemophagocytic syndrome (LAHS) [24].

Despite several efforts, only a few studies are currently available that have examined the role of sIL-2R and TSGF in PTC. In this study, we explored the clinical value of these two markers as indicators of effective treatment of PTC.

## 2. Materials and Methods

### 2.1. Patients

This study was a single-center, retrospective study. In total, 206 patients with PTC from Shanxi Province Cancer Hospital were enrolled between 20 June 2021 and 10 December 2021. All cases underwent surgery and were subsequently confirmed to be PTC by pathologists. The present study was approved by the Medical Ethics Committee of Shanxi Province Cancer Hospital.

The inclusion criteria were as follows: (1) histological confirmation of PTC, (2) patients who had not received any treatment before recruitment, such as surgery, chemotherapy, radiation, or other cancer-related treatments, and (3) patients with complete medical information. The following exclusion criteria were applied: (1) patients with other histological types of thyroid cancer, (2) patients who had received any thyroid cancer-related treatment before recruitment, (3) patients whose diagnosis was not confirmed by pathology, and (4) patients with incomplete medical information.

### 2.2. Data Collection

Epidemiological and clinical data were collected from patients' medical records including pathological diagnosis, age at diagnosis, sex, tumor size, lymphatic metastasis, focus, nodular goiter, Hashimoto thyroiditis (HT), and capsule invasion. Additionally, we obtained information from the immune function assay including serum levels of sIL-2R and TSGF before surgery from medical records.

### 2.3. Serum sIL-2R and TSGF Assay

A 3 mL sample of peripheral blood was withdrawn from PTC patients and serum was obtained after centrifugation at 1,000 × *g* for 10 minutes. Serum sIL-2R concentration was detected by ELISA (Human sIL-2R*α*/CD25 ELISA kit, MultiSciences (Lianke) Biotech, Co., Ltd., China) according to the manufacturer's instructions, and serum TSGF levels were measured using Automatic Biochemistry Analyzer.

### 2.4. Statistical Analysis

All data were analyzed using SPSS 22.0 software (IBS SPSS, Armonk, NY, USA). The optimal cut-off values for serum sIL-2R and TSGF levels were determined by receiver operating characteristic (ROC) analysis. The associations of the two predictors with the clinicopathological characteristics of PTC were analyzed using the chi-square test. Univariate and multivariate analyses were performed on the basis of the logistic regression model. *P* values < 0.05 were considered statistically significant.

## 3. Results

### 3.1. Basic Characteristics of Patients

A total of 206 patients with PTC were enrolled in our study. The ratio of males to females was 1 : 4.1. The average age of all cases was 45.81 years, with a median age of 45.5 years, ranging from 17 to 73 years. It is widely accepted that the increase in the incidence of thyroid cancer is due to the high detection of PTC, especially for papillary thyroid microcarcinoma (PTMC). PTC with a smaller tumor size (≤1 cm) represented 78.64%. Furthermore, PTC presents aggressive malignant characteristics such as multifocality, lymph node imvolvement, and capsular invasion. Of the 206 patients, 80 developed LNM, 87 had multifocal disease, and 70 had capsular invasion. In general, HT and nodular goiter were the most common thyroid diseases. Considering the high incidence of these diseases, 64.08% of patients with PTC had nodular goiter, while 19.41% had HT (Table 1).

### 3.2. Associations between Serum sIL-2R and TSGF Levels and Relevant Clinicopathological Characteristics in Patients with PTC

**Figure 1:**
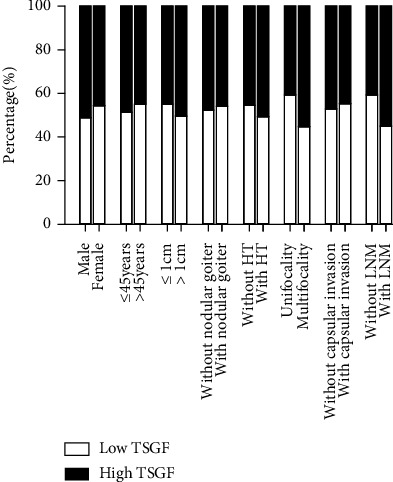
The relationship of serum TSGF level with clinicopathological parameters in patients with PTC.

**Figure 2:**
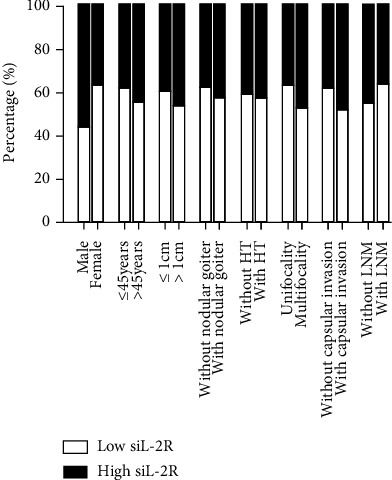
The relationship of serum sIL-2R level with clinicopathological parameters in patients with PTC.

We performed ROC analysis and determined that the cut-off values of serum TSGF and sIL-2R were 63.35 U/mL and 507 U/mL, respectively. Consequently, patients were divided into low-level and high-level groups for correlation analysis (Table 2). Serum TSGF was associated with focality (*χ*^2^ = 4.97, *P* = 0.026) and lymphatic metastasis (*χ*^2^ = 4.154, *P* = 0.042). The proportion of patients developing lymphatic metastases in PTC with high levels of TSGF was 55% compared to the 45% in patients with low TSGF (Figure 1). Similarly, multiple foci accounted for 55.2% of patients with highlevel of TSGF, which was superior to that of the low group. There was no significant association between TSGF level and age, gender, HT, nodular goiter, tumor size, or capsular invasion (*P* > 0.05). With regard to serum sIL-2R, sIL-2R levels were only correlated with gender (*χ*^2^ = 4.464, *P* = 0.035) (Figure 2). Furthermore, serum sIL-2R did not appear to have a significant correlation with other parameters (*P* > 0.05). Our findings support an association of immune-related factors with the malignant features of PTC.

### 3.3. Serum TSGF Served as an Independent Predictor of Lymph Node Involvement for Patients with PTC

**Figure 3:**
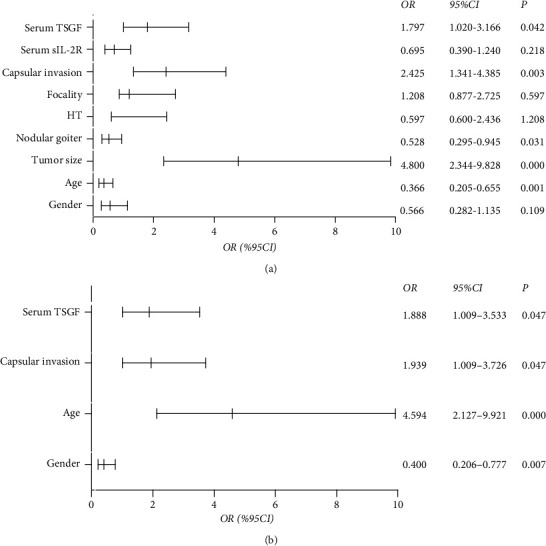
The univariate (a) and multivariate (b) logistic regression analysis.

To further elucidate the predictors for lymphatic metastasis in PTC, we used a logistic regression model to evaluate associated factors. Univariate logistic regression analysis indicated that age (OR: 0.366, 95% CI: 0.205-0.655, *P* = 0.001), tumor size (OR: 4.8, 95% CI: 2.344-9.828, *P* = 0.000), serum TSGF level (OR: 1.797, 95% CI: 1.02-3.166, *P* = 0.042), capsule invasion (OR: 2.425, 95% CI: 1.341-4.385, *P* = 0.003), and nodular goiter (OR: 0.528, 95% CI: 0.295-0.945, *P* = 0.031) were factors affecting LNM of PTC (Figure 3(a)). Multivariate regression analysis revealed that age > 45 years was a protective factor (OR: 0.4, 95% CI: 0.206-0.777, *P* = 0.007). In contrast, a larger tumor size (OR: 4.594, 95% CI: 2.127-9.921, *P* = 0.000), high serum levels of TSGF (OR: 1.888, 95% CI: 1.009-3.533, *P* = 0.047), and capsular invasion (OR: 1.939, 95% CI: 1.009-3.726, *P* = 0.047) could result in an increased risk of exposure to LNM in patients with PTC (Figure 3(b)).

## 4. Discussion

In the present study, we evaluated the predictive value of preoperative serum levels of sIL-2R and TSGF for LNM and evaluated their clinical implications in PTC. We found that both immune-related parameters were correlated with specific malignant characteristics of PTC. Serum sIL-2R levels were associated with gender (*P* < 0.05). TSGF level correlated with focality and LNM, and its predictive value for LNM was evaluated in our cohort.

sIL-2R is a marker of activated peripheral blood T cells. It plays an important role in several conditions such as malignancy, autoimmune diseases, inflammatory diseases, infections, and transplantation or rejection [16]. Currently, the contribution of sIL-2R to cancer development has been extensively explored. Mariotti et al. reported that serum sIL-2R was positively correlated with FT3 or FT4 levels, regardless of the autoimmune or nonautoimmune nature of the underlying hyperthyroid disease [17]. Circulating slL-2R seems to be strictly dependent on thyroid status in patients free of disease. Interestingly, there was elevated serum sIL-2R in a portion of patients with metastatic DTC after L-T4 therapy, despite the hypothyroid state [18]. Thyroxine suppressive treatment in patients may increase the serum level of sIL-2R because thyroid hormones can inversely modulate the cell-mediated immune response [19]. High-frequency ultrasound combined with the detection of serum high mobility group box (HMGB-1), sIL-2R, and thyroglobulin antibody level (TgAb) has diagnostic power in thyroid cancer (sensitivity: 98.0% and specificity: 95.0%) [25]. However, there was limited research currently available on the relationship between serum sIL-2R and PTC. Herein, we described serum sIL-2R levels in a cohort of 206 PTC patients. Our study found that sIL-2R levels were closely correlated with gender. Specifically, our cohort included 166 female patients, representing 80.58% or almost four times that of male patients. Among the female cases, low sIL-2R group accounted for 63.6%, which was significantly higher than the proportion in male patitents (45.0%). Of note, sIL-2R was correlated with clinicopathological parameters such as histological type, clinical stage, and tumor grade of ovarian cancer [20]. In our study, we did not find similar correlations except for the association with gender, which is indicative of the unclear role and underlying mechanism of sIL-2R in PTC.

TSGF is a well-known growth factor involved in the initiation of cancer and in the spread of cancer, facilitating the proliferation of peripheral capillaries in tumors and their surrounding tissues. Previous studies have revealed that serum TSGF levels have an excellent clinical value for early diagnosis [2–5, 9, 11], prognosis [6–8, 10, 13, 15], and response to the therapeutic outcomes [12, 14] of various types of tumors. In most studies, combination of serum TSGF with traditional biomarkers or other detection tools improved diagnostic precision [3]. TSGF levels have been associated with pancreatic cancer tumor differentiation [6] and LNM of colon cancer [7]. However, its role in PTC for predicting LNM had not been elucidated. Our results revealed that serum levels of TSGF correlated with focality in patients with PTC. To date, one clinical study showed that multifocality in thyroid cancer can be detected in 18-87% of cases [26]. In our study, 42.23% (87/206) of patients with PTC had multifocal disease which was consistent with the incidence reported previously [27, 28]. Geron et al. indicated that 534 of 1039 (51.4%) PTC patients harboring multifocal disease constituted a subgroup characterized by older age, male sex, more extrathyroidal extension, additional lymph node metastases, advanced TNM stage (stage III/IV), and increased risk of American Thyroid Association recurrence [27]. Kim et al. reviewed 672 of 2095 (32.07%) PTC patients with multifocal disease and found a correlation with an increased risk of disease recurrence/persistence [28]. Joseph et al. determined that multifocality was associated with an increased risk of developing LNM in 12 studies, indicating that multifocality in thyroid cancer served as a prominent risk factor for disease progression and recurrence [29]. Similarly, Kim et al. also found that multifocality showed a marked correlation with an elevated risk of recurrence of PTC on analyzing a total of 33,976 patients from 26 studies [30]. Herein, patients with high TSGF levels, which accounted for 55% of the study sample, had more multifocal disease, than patients with low TSGF. In contrast, the low TSGF group was prone to develop solitary PTC. Thus, an increase in serum TSGF may promote the appearance of multifocality and the progression of PTC.

We also found that lymphatic metastasis acted as the prevalent aggressive features in PTC. Despite decrease in mortality, LNM occurs frequently in young patients with PTC [31]. Liu et al. demonstrated that 44.5% of cases of PTC (21428/48166) developed LNM according to data from the Surveillance, Epidemiology, and End Results (SEER) database (2004-2015) and indicated that male sex, large tumor size, extrathyroidal extension, multifocality, and distant metastases were risk factors for LNM of PTC [31]. Min et al. determined that a total of 98 of 214 (45.8%) PTC patients presented central lymph node (CLN) metastasis [32]. In our present study, 38.83% of PTC patients exhibited lymphatic metastases. This was the first report to describe the relationship between serum TSGF levels and lymphatic metastasis in PTC. Specifically, the rate of lymphatic metastases in patients with PTC with a high level of TSGF was 55%, which prominently exceeded cases with a low level of TGSF. Thus, higher levels of TSGF may enhance lymphatic metastasis in PTC.

To date, a growing number of studies have identified prognostic indicators of LNM in PTC due to its potential risk of the disease progression. For example, Min et al. showed that the four risk factors could promote CLN metastasis for PTC patients with HT by the multivariate analysis. These factors included an increase in serum TgAb and sonographic characteristics such as lower tumor location, irregular CLN margin, and microcalcification. Subsequently, an individualized nomogram with a favorable C-index of 0.815 was established based on the four indicators for the management of CLN metastasis in clinical practice [32]. Yu et al. established a transfer learning radiomics (TLR) model to achieve reliable predictive performance of LNM in patients with PTC in the test set and in two independent testing sets, with an AUC of 0.93 compared to previous models. Their model included a statistical model (SM), traditional radiomics model (RM), and a nontransfer learning radiomics (NTLR) [33]. Importantly, Joseph et al. also found that LNM rates were negatively associated with age at diagnosis in patients with PTC aged 18-59 years, implying that age at diagnosis decreased the risk of LNM in patients with PTC (OR = 0.974, 95% CI: 0.972-0.975, *P* < 0.0001) [29]. Zhao et al. revealed that younger age, a normal body mass index, BRAF^V600E^ mutation, larger maximum diameter, left lobe tumor, aspect ratio > 1, capsular invasion, and calcification were significant risk factors for central lymph node metastasis in PTC patients with HT [34]. Our model analysis revealed that the young age at diagnosis, larger tumor size, nodular goiter, capsular invasion, and higher serum TSGF level may be the risk factors for LNM of PTC. Multivariate analysis showed that younger age, tumor size, capsular invasion, and increased TSGF levels were factors that associated with LNM in patients with PTC. However, serum sIL-2R levels did not appear to have a significant predictive role for LNM in patients with PTC, given its lack off correlation with clinical parameters. Overall, our results suggest that TSGF may act as a promising candidate marker for lymphatic metastasis in PTC cases. Our findings shed light on the clinical utility of TSGF in cancer.

## 5. Conclusions

Taken together, our findings strongly support the role of serum TSGF in PTC progression. Furthermore, our findings highlight the role of serum TSGF as a prognostic indicator of LNM in PTC. Nonetheless, serum sIL-2R levels had an inferior association with PTC, although its levels were associated with gender. There were some limitations to our study based on the limited sample size. A large-scale multicenter cohort study will be conducted to provide support for the clinical utility of TSGF in identifying LNM in patients with PTC. Further studies will attempt to elucidate the molecular mechanisms underlying TSGF activity in PTC to provide stronger evidence supporting its application in clinical practice.

## Figures and Tables

**Table 1:** The clinical characteristics of PTC patients.

Characteristics	*n*
Gender	
Male/female	40/166
Age	
≤45 years/>45 years	103/103
Tumor size	
≤1 cm/>1 cm	162/44
LNM	
No/yes	126/80
Focality	
Unifocality/multifocality	119/87
Capsular invasion	
No/yes	136/70
Nodular goiter	
No/yes	74/132
HT	
No/yes	166/40

**Table 2:** The relationship between serum sIL-2R and TSGF level and clinicopathological parameters in patients with PTC.

	sIL-2R				TSGF			
	Low	High	*χ* ^2^	*P*	Low	High	*χ* ^2^	*P*
Gender								
Male	18 (45.0)	22 (55.0)	4.464	0.035^∗^	20 (50.0)	20 (50.0)	0.301	0.583
Female	105 (63.6)	61 (36.4)			91 (54.8)	75 (45.2)		
Age								
≤45 years	65 (63.1)	38 (36.9)	0.989	0.32	54 (52.4)	49 (47.6)	0.176	0.675
>45 years	58 (56.3)	45 (43.7)			57 (55.3)	46 (45.7)		
Tumor size								
≤1 cm	99 (61.1)	63 (38.9)	0.62	0.431	89 (54.9)	73 (45.1)	0.34	0.56
>1 cm	24 (54.5)	20 (45.5)			22 (50.0)	22 (50.0)		
Nodular goiter								
No	47 (63.5)	27 (37.5)	0.695	0.404	39 (52.7)	35 (47.3)	0.065	0.799
Yes	76 (57.6)	56 (42.4)			72 (54.5)	60 (45.5)		
HT								
No	100 (60.2)	66 (39.8)	0.101	0.751	91 (54.8)	75 (45.2)	0.301	0.583
Yes	23 (57.5)	17 (42.5)			20 (50.0)	20 (50.0)		
Focality								
Unifocality	76 (63.9)	43 (36.1)	2.024	0.155	72 (60.5)	47 (39.5)	4.97	0.026^∗^
Multifocality	47 (54.0)	40 (46.0)			39 (44.8)	48 (55.2)		
Capsular invasion								
No	86 (63.2)	50 (36.8)	2.069	0.15	72 (52.9)	64 (47.1)	0.143	0.705
Yes	37 (52.9)	33 (47.1)			39 (55.7)	31 (44.3)		
LNM								
No	71 (56.3)	55 (43.7)	1.522	0.217	75 (59.5)	51 (40.5)	4.154	0.042^∗^
Yes	52 (65.0)	28 (35.0)			36 (45.0)	44 (55.0)		

^∗^ *P* < 0.05.

## Data Availability

The data used to support the findings of this study are available from the corresponding author upon reasonable request.
